# Female mice lacking ERβ display excitatory/inhibitory synaptic imbalance to drive the pathogenesis of temporal lobe epilepsy

**DOI:** 10.7150/thno.56331

**Published:** 2021-04-07

**Authors:** Zhongke Wang, Ruxin Xie, Xiaolin Yang, Huachun Yin, Xin Li, Tianyao Liu, Yuanyuan Ma, Junwei Gao, Zhenle Zang, Ruotong Ruan, Yang Li, Kaixuan Huang, Qingbo Chen, Kaifeng Shen, Shengqing Lv, Chunqing Zhang, Hui Yang, Maragret Warner, Jan-Ake Gustafsson, Shiyong Liu, Xiaotang Fan

**Affiliations:** 1 Department of Neurosurgery, Xinqiao Hospital, Army Medical University (Third Military Medical University), 400037 Chongqing, China.; 2 Department of Developmental Neuropsychology, School of Psychology, Army Medical University (Third Military Medical University), 400038 Chongqing, China.; 3 Center for Nuclear Receptors and Cell Signaling, University of Houston, Houston, TX 77054;; 4 Center for Innovative Medicine, Department of Biosciences and Nutrition, Karolinska Institute, 141 86 Novum, Sweden.

**Keywords:** ERβ, temporal lobe epilepsy, estrogen, hippocampus, synapse

## Abstract

Epilepsy is a highly prevalent and drug-refractory neurological disorder characterized by spontaneous recurrent seizures. Estrogen is identified to be proconvulsant and lowers the seizure threshold of female epilepsy. Estrogen receptor β (ERβ) has been proposed to mediate neuroprotection in epilepsy, although the underlying mechanism remains unknown.

**Rationale:** In this study, we investigated the role of ERβ in the epileptogenesis of female temporal lobe epilepsy (TLE).

**Methods:** Immunohistochemistry, immunofluorescence, western blots, Golgi staining, ^1^H MRS and whole-cell patch-clamp were used to evaluate ERβ expression, pathological changes, and synaptic excitation /inhibition (E/I) balance in female TLE patients and ovariectomized (OVX) chronic epileptic mice. Electroencephalogram (EEG) recordings were recorded to evaluate the epileptic susceptibility in OVX WT and ERβ^-/-^ mice. And high-throughput RNA-sequence was performed to identify differential expression genes (DEGs) which can elucidate the potential mechanism of ERβ regulating the seizure susceptibility.

**Results:** ERβ expression was decreased in the brains of female TLE patients and OVX chronic epileptic mice. ERβ deletion enhanced seizure susceptibility and exacerbated the imbalance of synaptic E/I in hippocampal CA1 area of OVX epileptic mice. In line with these observations, RNA-sequence data further identified glutamine ligase (GLUL) as the target of ERβ involved in regulating synaptic E/I in CA1. Furthermore, ERβ agonist WAY-200070 markedly suppressed epileptic phenotypes and normalized GLUL expression in CA1 region of kainic acid (KA) induced OVX chronic epileptic model.

**Conclusions:** Our data provide novel insight into the pathogenesis of female TLE, and indicate ERβ provides a new therapeutic strategy for female TLE patients.

## Introduction

Epilepsy is a serious neurological disease typified by spontaneous recurrent seizures (SRS), which affects more than 50 million individuals worldwide. Approximately one third of patients with epilepsy remain antiepileptic drug (AED) resistant, and temporal lobe epilepsy (TLE) is the most common drug-resistant epilepsy 1, 2. Female patients with epilepsy present a unique management challenge because hormonal level fluctuation is involved in epileptic seizures 3. Epileptic seizures become more frequent in female patients with increased estrogen levels and ratio of estrogen to progesterone. And estrogen is identified to be proconvulsant and lowers the seizure threshold of female epilepsy 4, 5. Rodent studies have suggested exogenous estrogen to have no effect or to facilitate or to inhibit seizure activity and seizure‐induced damage 6, 7. Additional studies are required to determine exact roles of estrogen in pathogenesis of epileptic seizures.

Estrogenic actions are mediated mainly through estrogen receptors (ERα and ERβ), and both ERα and ERβ show differential expression in the central nervous system (CNS) 8. ERα is abundantly expressed in ventromedial hypothalamus and pituitary, controlling reproduction, while ERβ influences nonreproductive processes in the brain. ERβ is the main ER expressed in the cerebral cortex and hippocampus, where epilepsy commonly occurs 9, 10. It has been confirmed that selective estrogen receptor modulators with activity at ERβ, but not ERα, produce antiseizure effects 11. Notably, one recent study has indicated that an ERβ agonist, displayed protection against seizure-induced oxidative brain injury and associated memory dysfunction 12. ERβ deficit may be potentially linked with female epilepsy, however, the underlying mechanism is not well understood.

Synaptic excitatory/inhibitory (E/I) imbalance has been implicated as a cause of epileptogenesis 13. Previous study had reported intracortical excitability of humans was increased in the late-follicular phase when estradiol concentration was high 14. Estrogen could enhance neuronal excitability during the proestrus phase relative to the metestrus phase of the rat estrous cycle, and potentiate neuronal excitability by activating NMDA receptor and affecting GABAergic metabolism in female mice 15, 16. Moreover, an ERβ ligand (LY3201) was reported to increase glutamic acid decarboxylase (GAD) expression in layer V of cortex and CA1 of hippocampus and decrease glutamate receptor expression in layer V of cortex 17. Recently, we further confirmed that ERβ deletion reduced GABAergic signaling in the primary motor cortex of mice 18. These observations suggested that high level of estrogen results in a synaptic E/I imbalance that could explain the epileptic phenotype, and the protection of ERβ in epilepsy might be related to rescue synaptic E/I imbalance.

In this study, we first reported the expression of ERβ, but not ERα, was down-regulated in epileptic brain tissues from female TLE patients and ovariectomized (OVX) chronic epileptic mice. ERβ deletion increased the susceptibility to seizure, exacerbated the pathological changes of epilepsy and aggravated the synaptic E/I imbalance of CA1 in OVX chronic epileptic mice. Further, RNA-sequence revealed Glutamine ligase (GLUL) may participate in the regulation of ERβ on neuronal excitability of CA1. Finally, ERβ agonist WAY-200070 (WAY) alleviated epileptic seizures and rescued downregulated GLUL expression in OVX chronic epileptic mice. These findings provide evidence of ERβ as a mediator of female TLE epileptogenesis.

## Methods

### Human brain tissue specimens

The human brain tissues were obtained from the Department of Neurosurgery at Xinqiao Hospital (Army Medical University, Chongqing, China). The project had ethical approval (through Medical Ethics Committee of Xinqiao hospital 092-01/2020). Patients underwent comprehensive presurgical evaluation, such as neurological examination, intelligence assessment, MRI/PET-CT scans, and EEG recordings, and signed informed consents for use of resected brain tissues in research before surgery. Control brain tissues were obtained from autopsies. Detailed clinical information from patients and controls are summarized in Table S1 and Table S2 respectively. In addition, to avoid the effect of estrogen levels on ERβ, all specimens were collected within three days after menstruation to ensure the serum estrogen level was as consistent as possible.

The brain tissues were divided into two parts. One part was fixed in 4% paraformaldehyde (PFA) for 24-48 h and embedded in paraffin to make 5 μm tissue sections. Paraffin sections were used for histopathologic diagnosis and IHC. Another part was stored in liquid nitrogen and used to extract protein for western blot analysis.

### Animals

Age-matched 6-week-old female WT and ERβ knockout (ERβ^-/-^) mice were housed in the controlled environment with a 12 h light/12 h dark illumination schedule, standardized temperature at 25 °C and available food and water ad libitum. All experimental procedures were performed according to the principles of Care and Use of Laboratory Animals approved by National Institutes of Health Guide and the laboratory animal ethical principles approved by the Army Medical University. All mice in this study were ovariectomized at 6 weeks of age and allowed to recover for one week according to our previous description 19. Mice were injected with castor oil containing β-estradiol (30 μg/kg/day, s.c.) after recovery to maintain the physiological estrogen level until they were sacrificed 20.

### Electrodes implant for electroencephalogram (EEG) recording

EEG recordings started at 8-week-old OVX mice (weight range: 20-25 g) according to previous study 21. Briefly, OVX mice were anesthetized with isoflurane, and implanted with injection guide cannula and recording electrodes under stereotaxic guidance. Cortical electrodes were implanted into left frontal and two parietals respectively, along with a ground electrode positioned over the nasal sinus. Depth electrode was implanted into the left dorsal hippocampus [from bregma (mm): nose bar 0; anteroposterior: -1.80; mediolateral: -1.60; dorsoventral: -1.60 below dura mater] with an injection cannula glued to depth electrodes for intrahippocampal kainic acid (KA) injection. The electrodes together with cannula were secured to the skull by super glue, and animals were allowed a week to recover. Electrodes were connected the EEG100C amplifier through bank cable to perform EEG recordings, and the signals were filtered (1 to 500 Hz) and digitized at 3 kHz. Time-frequency analysis and normalized power of EEG recordings [delta (δ), theta (θ), alpha (α), beta (β), lower gamma (γ1), higher gamma (γ2), ripple and fast ripple (fripple)] were performed in the custom code and brainstorm based on Matlab 2017.

### KA-induced epileptic models in OVX mice

OVX acute seizures and chronic epileptic mouse models were established by unilateral intrahippocampal injection of KA. This model was reported to recapitulate the major epileptiform and neuropathological features of human epilepsy. OVX mice unilaterally intrahippocampally injected with normal saline (NS) were defined as controls.

Acute epileptic model was induced by left intrahippocampal injection (anteroposterior: -1.60; mediolateral: -1.50; dorsoventral: -1.60) of KA (7 ng in 0.5 μl NS, 0.5 μl min^-1^) using a 0.5 μl Hamilton syringe in 9-week-old OVX mice. At the end of injection, syringe was maintained *in situ* for an additional 5 min to limit the reflux of KA. Basal EEG recordings were done before KA injection, and epileptic seizures of OVX mice were terminated 2 h after KA injection by using diazepam. OVX acute epileptic mice were sacrificed when at least 30 min EEG recordings similar with baseline were monitored. Seizure behavioral scores, average latency, number, and time of seizures before status epilepticus (SE) were recorded and analyzed. Seizure behavioral scores were evaluated according to Racine scale 22.

Chronic epileptic model was induced by the left unilateral intrahippocampal injection (anteroposterior: -1.60; mediolateral: -1.50; dorsoventral: -1.60) of KA (200 ng in 50 nl NS, 0.5 μl min^-1^) in 9-week-old OVX mice. SRSs could be recorded after SE lasting for about 3 h, and were stable, reproducible, and not clustered. Besides, the histopathological features of chronic epileptic model were similar with human epilepsy, such as gliosis, neuronal loss, and mossy fiber sprouting. All OVX chronic epileptic mice used in this study were recorded to SRSs before 12 weeks old. Seizure behavioral score, time of spontaneous seizures onset and frequency and duration of spontaneous seizures were evaluated.

### Immunohistochemistry (IHC) and immunofluorescence (IF)

According to our previous method 23, the brain tissues from humans and mice were processed for paraffin or cryostat sections. For paraffin sections, sections (5 μm thick) were collected, deparaffinized, rehydrated and processed for antigen retrieval. For cryostat sections, coronal cryosections (30 μm) were collected. Then paraffin and cryostat sections were incubated in 3 % H2O2, 0.3% Triton X-100 and 3% bovine serum albumin (BSA) in turn to eliminate the endogenous peroxidase and block nonspecific binding. Next, sections were incubated with anti-ERβ (1:200; from the Jan-Ake Gustafsson's laboratory, Karolinska Institute, Novum, Sweden), anti-VGAT (1:400, SYSY), anti-VGluT1 (1:400, SYSY) and anti-GFAP (1:1000, Millipore) in antibody diluent overnight at room temperature. To verify the specificity of antibodies, PBS was used instead of primary antibodies in the negative controls. After washing with 0.01M PBS, sections were incubated with the secondary antibodies in 1:200 dilutions for 2 h at 37 °C, the avidin-biotin-peroxidase complex for 2 h at 37 °C and 3,3-diaminobenzidine tetrahydrochloride to staining the target protein. For immunofluorescence, sections were incubated with Cy3-or 488-conjugated (both at 1:500, Jackson ImmunoResearch) (2 h, 37 °C) as secondary antibodies, then sections were incubated with 4',6-diamidino-2-phenylindole DAPI (Sigma-Aldrich, St. Louis, MO) to redye. Stained specimens were observed and captured using a Zeiss Axivert microscope (Oberkochen, Germany) equipped with a ZeissAxioCam digital color camera connected to the Zeiss AxioVision 3.0 system.

The immunoreactivity of IHC was quantified using Image J software and based on the mean optical density (MOD) in individual cell 24. Values of optical density (OD) in stained specimens (three slides for each specimen) were calculated by the equation: ∑ Integral optical density (IOD) / ∑ Area, and the value of MOD was determined for each specimen. ∑ IOD is the sum of integral optical density of all cells in the photograph, and ∑ Area is the total area of all cells in the photograph 24. And the immunoreactivity of IF was quantified based on the fluorescence intensity.

### Nissl staining

Frozen sections were fixed in 4% PFA for 10 min at room temperature and washed in distilled water. Then the sections were stained with Nissl staining solution (Beyotime, Jiangsu, People's Republic of China) for 20 min at 37 °C. Next, they were dehydrated in 95% ethanol, immersed in xylene, and mounted in DPX (06522, Sigma, United States). Images of the Nissl staining were captured under 40X objective lens and Nissl positive cells were analyzed.

### Timm staining

Mice were deeply anesthetized and perfused, and brain tissues were removed and fixed according to the user manual of FD Rapid Timm Stain Kit. Coronary sections (30 μm) were cut, stained, rinsed, dehydrated, cleared, and mounted in DPX in turn. Images of the Timm staining were captured under 40X objective lens and Timm scores were evaluated on a scale of 0-5 according to previous method 25.

### Golgi staining

Mice were decapitated and fresh brains were dissected immediately. The brains were processed, sectioned coronally and stained following the guide of FD Rapid Golgi Stain Kit. Then the coronary sections (100 μm) were sealed in DPX and images of sections were captured under 20X and 100X oil objective lens. Sholl analysis was used to evaluate the dendrite complexity. Dendrite spine density and dendrite complexity were analyzed using the ImageJ software.

### Western blot

The protein of brain tissues from human and mouse were extracted, and protein concentration was measured using a BCA protein assay. Extracted protein was mixed with 5× loading buffer, electrophoresed in 10% sodium dodecyl sulfate polyacrylamide gel, and transferred onto a polyvinylidene difluoride membrane. The membranes were immersed in 5% skim milk for 2 h at room temperature. After washing with TBST, the membranes were incubated overnight at 4 °C with the primary antibodies. The primary antibodies used in this study were as follows: anti-GAPDH (1:1000, Abcam), anti-ERβ (1:500; from the Jan-Ake Gustafsson's laboratory, Karolinska Institute, Novum, Sweden), anti-VGAT (1:1000, Abcam), anti-VGluT1 (1:1000, Abcam), and anti-Glutamine Synthetase (1:1000, Abcam). After washing, the membranes were incubated with the anti-rabbit or anti-mouse secondary antibodies for 2 h at room temperature. The specific protein bands were visualized in membranes by the chemiluminescence method (Amersham, Piscataway, NJ), and the optical density of protein bands were analyzed in Bio-Rad Image-Lab 6.0 software.

### Quantitative real-time polymerase chain reaction (qRT-PCR)

Total RNA in CA1 of OVX mice was extracted using TRIzol (Invitrogen, Carlsbad, CA, USA), and the concentration of the RNA was determined using a spectrophotometer (OceanOptics, Dunedin, FL). Then, extracted RNA was reverse transcribed to cDNA. The primers of target DNA were synthesized and the detailed sequences of primers used in this study were described in supplementary Table S3. GAPDH was regarded as control gene. The amplification conditions of RT-PCR were as follows: 95 °C for 30 s (1 cycle), 95 °C for 5 s (40 cycles) and 60 °C for 30 s (1 cycle). The analysis of RT-PCR was performed according to the 2^-ΔΔCT^ method.

### Proton magnetic resonance spectroscopy (^1^H MRS)

Human ^1^H-MRS data were acquired from the Radiology Department of Xinqiao hospital. The ^1^H MRS of human hippocampus were performed using a 3.0 T medical system (General Electric Medical Systems), and the detailed parameters of the imaging sequence were as following: slice thickness: 20 mm; repetition time (TR)/echo time (TE) = 1500/30 ms; voxel size: 15 × 15 × 20 mm^2^; field of view (FOV): 240 × 240 mm^2^; phase: 16; data points = 2048. The ^1^H MRS of animal hippocampus were performed on a Bruker 7.0 T MR scanner (Bruker, Ettlingen, Germany). 16-week-old OVX mice were placed in the cradle of scanner and anesthetized with isoflurane throughout the scan. The T2-weighted anatomical images were scanned first by a standard spin echo multi-slice (SEMS) imaging sequence, the detailed parameters were as follows: TR/TE = 3000/40 msec, matrix size = 256 × 256, field of view (FOV) = 25 × 25 mm^2^. Next, the voxel of hippocampal region was chosen based on T2-weighted images for ^1^H MRS data acquisition. The detailed parameters were as follows: TR/TE = 2000/16.501 msec, voxel size = 1.5×1.5×1.5 mm^3^, spectrum width = 3301 Hz, complex points = 2048, and number of averages = 512. All MRS data were analyzed on the spectral range of 0.2-4.2 ppm by TARQUIN (version 4.3.10) and the creatine (Cr) as the reference peak due to Cr expression is stable in brain.

### Whole-cell patch-clamp recording

Sixteen-week-old OVX WT, epileptic, ERβ^-/-^ and ERβ^-/-^ epileptic mice were sacrificed, and their brains were dissected rapidly to prepare brain slices for the whole-cell patch-clamp recordings according to previous methods 26. Coronal brain slices (300 μm) were cut in the following slice solution: 2 mM CaCl_2_, 2 mM MgCl_2_, 2.5 mM KCl, 26 mM NaHCO_3_, 1.25 mM KH_2_PO_4_, 10 mM glucose, and 220 mM sucrose [pH 7.4] continuously bubbled with 95% O_2_/5% CO_2_. Then, the brain slices were transferred to the artificial cerebrospinal fluid (ACSF): 126 mM NaCl, 26 mM NaHCO_3_, 10 mM glucose, 3 mM KCl, 1.4 mM NaH_2_PO_4_, 2 mM CaCl_2_, and 1 mM MgCl_2_ [pH 7.35-7.40] continuously bubbled with 95% O_2_/5% CO_2_. For whole-cell patch-clamp recordings, the brain slices were initially perfused in flowing ACSF (4 ml/min).

For spontaneous excitatory postsynaptic current (sEPSC) recordings, patch pipettes with a resistance of 3-6 MΩ were filled with the internal solution: 120 mM potassium methane-sulfonate, 10 mM NaCl, 10 mM EGTA, 1 mM CaCl_2_, 10 mM HEPES, 5 mM ATP-Mg, pH adjusted to 7.2 with KOH, with an osmolarity of 300 mOsm. Recordings were performed at -70 mv holding potential in the presence of 50 μM picrotoxin. For spontaneous inhibitory postsynaptic currents (sIPSC) recordings, patch pipettes were filled with the following internal solution: 130 mM cesium methane-sulfonate, 10 mM sodium methanesulfonate, 10 mM EGTA, 1 mM CaCl_2_, 10 mM HEPES, 5 mM lidocaine N-ethyl bromide quaternary salt-Cl, 2 mM ATP-Mg, pH adjusted to 7.2 with CsOH, with an osmolarity of 300 mOsm. Recordings were performed at 10 mv holding potential in the presence of 20 μM CNQX and 50 μM AP5. Signals from the CA1 pyramidal neurons were acquired using a MultiClamp 700B amplifier (Axon, USA) and recorded using pClamp 9.2 software (Molecular Devices, Sunnyvale, CA, USA). The signals were filtered at 2 kHz, digitized at 10 kHz, and analyzed with Clampfit 10.0 software and Mini Analysis Program (Synaptosoft, Leonia, NJ, USA).

### High-throughput RNA-sequence

The hippocampal CA1 region from 16-week-old OVX WT, epileptic and ERβ^-/-^ and ERβ^-/-^ epileptic mice were microdissected for RNA-sequence assay. The RNA-sequence was performed by Novogene company (Beijing, China). First, the purity, concentration and integrity of RNA extracted from CA1 were assessed, and the sequencing libraries were generated on the basis of extracted RNA. Then the clustering and sequencing of the library preparations were processed, and Differential expression genes (DEGs) were identified using the DEseq2 package. Genes with an absolute fold-change (FC) > 1.2 and false discovery rate (FDR) < 0.05 were considered as DEGs. Gene ontology (GO) and Kyoto Encyclopedia of Genes and Genomes (KEGG) pathways were enriched to explore functional annotation of DEGs using the ToppGene server with an adj.p value < 0.05. To explore the protein-protein interaction (PPI) network, the list of DEGs was mapped to the STRING online database (confidence score cutoff > 700) using NetworkAnalyst web. Communities of network were detected by InfoMap algorithm and visualized by Cytoscape (v3.6).

### Statistical analysis

The Unpaired two-tailed *t* tests were used to compare differences between two groups and one-way ANOVA was used to compare differences among three or four groups. Spearman's rank correlation tests were carried out to evaluate the bivariate correlation analysis. Significances were reached at *P* < 0.05. The data were expressed as mean ± SEM, and analyzed by the SPSS 18.0 package (SPSS, Inc., Chicago, IL, USA).

## Results

### Decreased ERβ expression in the female epileptic cortex and hippocampus

**Figure 1:**
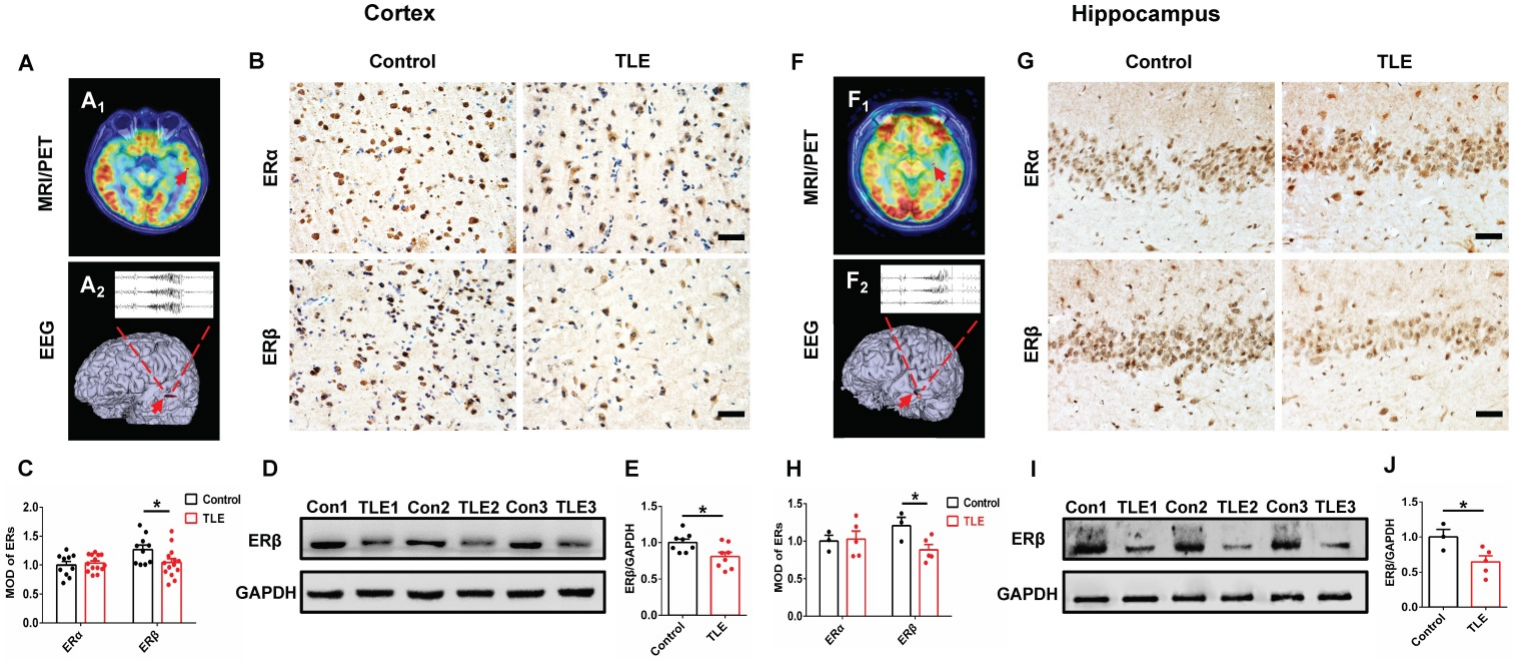
** ERβ expression in the temporal neocortex and hippocampus was downregulated in female TLE patients. (A**,** F)** Examples of preoperative localization for epileptogenic focuses in temporal neocortex (A) and hippocampus (F) of female TLE patients. **(B**,** G)** Immunostaining of ERα and ERβ in temporal neocortex (B) and hippocampus (G) of female TLE patients and controls. Scale bars, 50 µm. **(C**,** H)** Quantification of ERα and ERβ in immunostaining. MOD of ERβ was decreased in temporal neocortex (C; n = 14 in TLE, n = 10 in controls) and hippocampus (H; n = 5 in TLE, n = 3 in controls) of female TLE patients. There were no significant differences in the MOD of ERα. **(D**,** I)** Western blot of ERβ in temporal neocortex (D) and hippocampus (I) of female TLE patients and controls. **(E, J)** Western blot analysis showed that ERβ expression was decreased in temporal neocortex (E; n = 8 in TLE, n = 8 in controls) and hippocampus (J; n = 5 in TLE, n = 3 in controls) of female TLE patients compared with controls. The data were shown as means ± SEM. Significance was calculated using Student's *t* test. *P < 0.05.

To characterize a potential role for ERα or ERβ in the pathophysiology of epilepsy, the expression patterns were assessed in temporal neocortical and hippocampal brain tissues from female TLE patients and controls using IHC. MRI/PET-CT scans and EEG recordings were performed to ensure the epileptogenic focus from temporal neocortex (example is shown in Figure 1A) and hippocampus (example is shown in Figure 1F) of female patients with TLE. IHC showed that ERβ expression levels were significantly decreased in the temporal neocortex (Figure 1B-C) and hippocampus (Figure 1G-H) compared with controls. However, ERα expression did not reveal significant differences between female TLE patients and controls. When quantitated by western blot, decreased ERβ expression in the temporal neocortex (Figure 1D-E) and hippocampus (Figure 1I-J) was observed in female TLE patients compared with controls. In aggregate, Spearman rank correlation indicated a significant inverse correlation between ERβ expression and seizure frequency (Figure S1A-B; neocortex: *r* = -0.7776, *P* = 0.0231, hippocampus: *r* = -0.9307, *P* = 0.0217). Mental decline and cognitive impairment are the more frequent comorbidities associated with chronic epilepsy. Correlations with cognitive performance demonstrated positive correlations of ERβ expression and Wechsler intelligence scores (Figure S1C-D; neocortex: *r* = 0.7257, *P* = 0.0416, hippocampus: *r* = 0.8882, *P* = 0.0441).

Further, we checked whether there was decreased level of ERβ in an experimental OVX mouse model of chronic epilepsy induced by intra-hippocampus microinjection of KA. Indeed, we observed markedly reduced ERβ expression in the temporal cortex and hippocampus of 16-week-old OVX chronic epileptic mice compared with OVX WT mice by IHC (Figure S2A-B). It is possible that the down-regulation of ERβ may affect the epileptogenesis of female TLE.

### ERβ deletion increased susceptibility to KA-induced seizures in OVX mice

The role of ERβ in the susceptibility to seizures was investigated in control and KA-injected OVX WT and ERβ^-/-^ mice. We first determined whether ERβ affected acute seizures via EEG recordings for 2 h after KA injection in OVX acute epileptic mice (Figure S3A-C). ERβ deletion in OVX acute epileptic mice greatly increased θ, α, β oscillations in the cortex and θ, α, β, γ1 oscillations in the hippocampus compared with OVX WT mice (Figure S3D-E). Additionally, seizure behavioral score, average latency, number, and time of seizures before status epilepticus (SE) showed the susceptibility to epileptic seizures was increased in OVX ERβ^-/-^ mice compared with OVX WT mice (Figure S3F-I).

**Figure 2:**
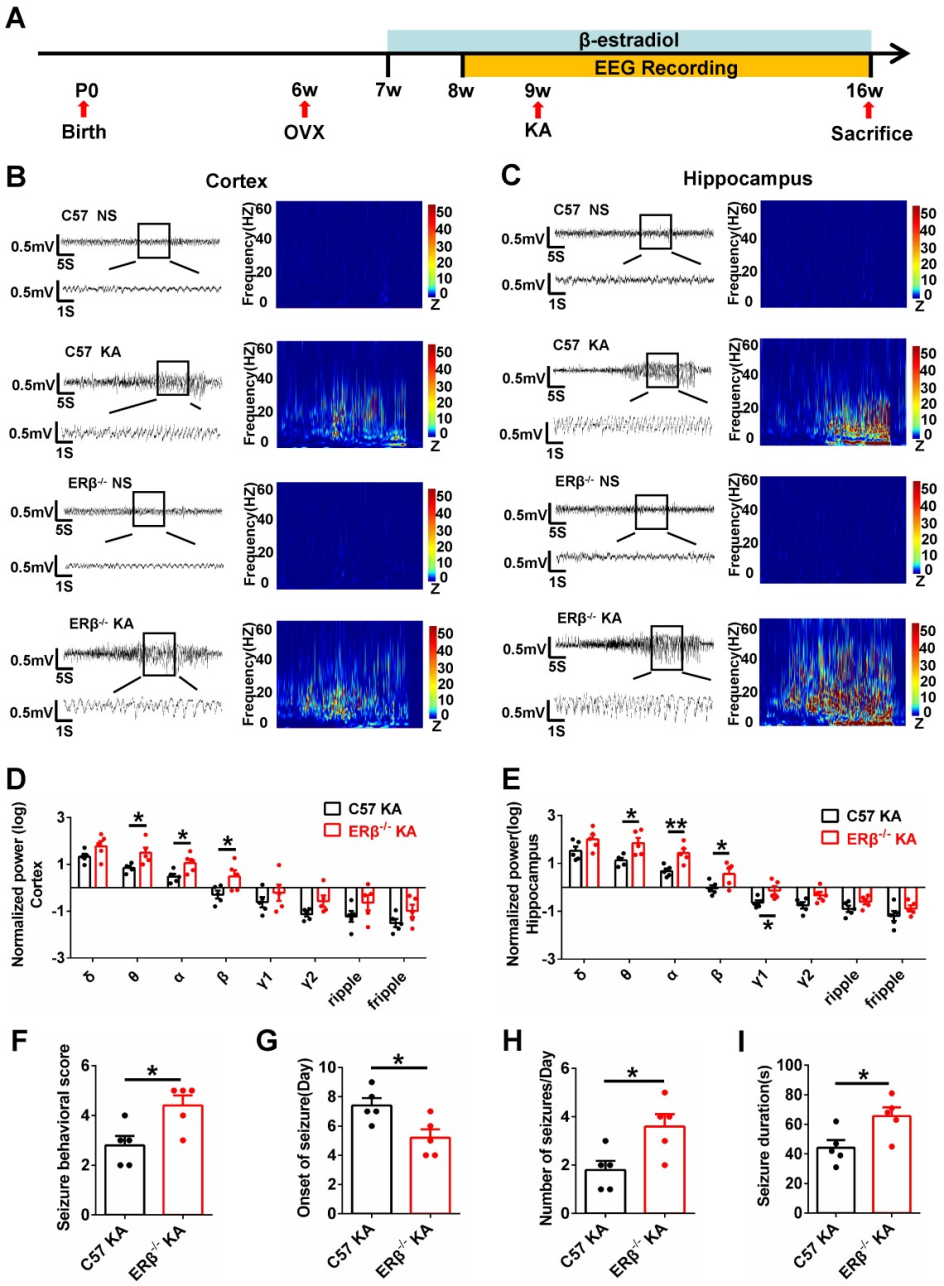
** ERβ deletion increased susceptibility to KA-induced seizures in OVX chronic epileptic mice. (A)** Diagram of experimental paradigm.** (B**,** C)** Representative baseline and chronic epileptic EEG recordings from the cortex (B) and hippocampus (C) of OVX mice.** (D**,** E)** Spectral analysis of chronic epileptic EEG recordings. ERβ deletion increased θ, α, β oscillations in cortex (D) and θ, α, β, γ1 oscillations in hippocampus (E) of OVX chronic epileptic mice. **(F**-**I)** Seizure behavioral score (F), onset of seizure (G), number of seizures per day (H) and seizure duration (I) of chronic epilepsy from OVX chronic epileptic mice. ERβ deletion increased the seizure behavioral score, frequency of seizures and seizure duration, and decreased the time of seizure onset. N = 5 in each group. The data were shown as means ± SEM. Significance was calculated using Student's *t* test. *P < 0.05, **P< 0.01.

The occurrence of SE is sufficient to induce SRS in diverse mammalian species, and many patients experienced an episode of continuous SE years prior to the onset of TLE 27. We next sought to verify whether ERβ affected the development of SRS. Mice underwent 8 weeks of video-EEG monitoring to evaluate SRS activity (Figure 2A). The baseline and spontaneous epileptic EEG traces from the cortex and hippocampus of OVX WT and ERβ^-/-^ mice were recorded (Figure 2B-C). ERβ deletion did not cause differences in the relative power of baseline EEG recordings from OVX WT mice. But ERβ deletion increased θ, α, β oscillations in the cortex (Figure 2D) and θ, α, β, γ1 oscillations in the hippocampus (Figure 2E) of OVX chronic epileptic mice. Further, we found ERβ deletion increased seizure behavioral score (Figure 2F), number of seizures per day (Figure 2H), seizure duration (Figure 2I), and decreased the time of SRS onset (Figure 2G) in OVX chronic epileptic mice.

Gliosis, neuronal loss and mossy fibers sprouting in CA1 and CA3 of hippocampus are prevalent pathological features of epilepsy 28. ERβ deletion exacerbated pathological changes in OVX chronic epileptic mice, and was reflected by GFAP immunostaining (Figure S4A), Nissl staining (Figure S4B) and Timm staining (Figure S4C). These results demonstrated that ERβ deletion increased seizure susceptibility in OVX chronic epileptic models.

### ERβ deletion exacerbated synaptic E/I imbalance in hippocampus

**Figure 3:**
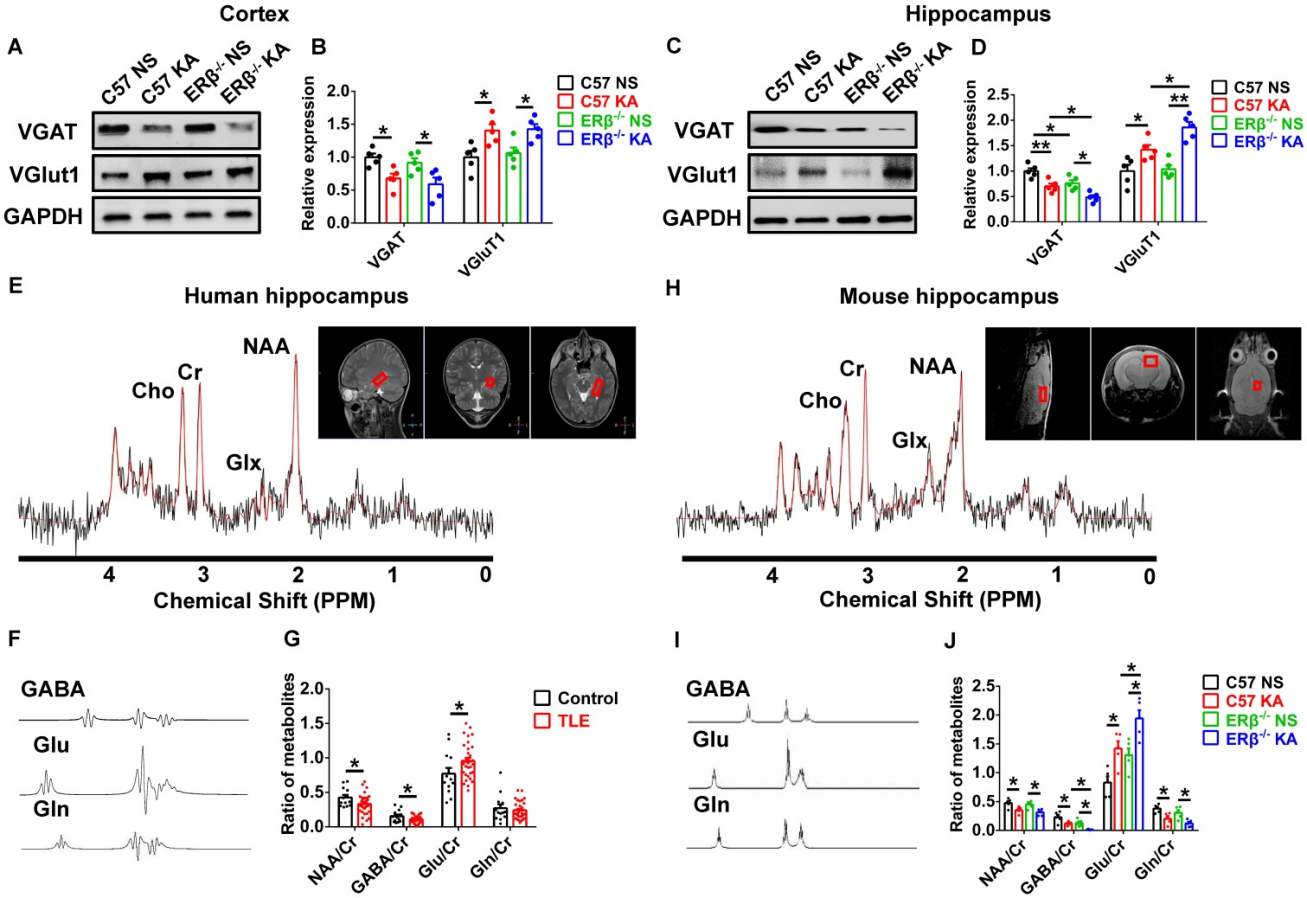
** ERβ deletion exacerbated synaptic E/I imbalance in hippocampus. (A**-**D)** Western blot analysis of VGAT and VGluT1 in cortex and hippocampus of OVX mice. VGAT expression was decreased and VGluT1 expression was increased in the cortex and hippocampus of OVX chronic epileptic mice. ERβ deletion especially decreased VGAT expression in hippocampus of OVX mice, and exacerbated decreased VGAT expression and increased VGluT1 expression in hippocampus of OVX chronic epileptic mice (n = 5 in each group). Data were shown as means ± SEM. Significance was calculated using one-way analysis of variance (ANOVA), followed by Tukey's test. **(E**-**G)** NAA/Cr and GABA/Cr were decreased and Glu/Cr was increased in hippocampus of female patients (n = 33 in TLE, n = 13 in controls). The data were shown as means ± SEM. Significance was calculated using Student's *t* test. **(H**-**J)** NAA/Cr, GABA/Cr and Gln/Cr were decreased and Glu/Cr was increased. ERβ deletion further decreased GABA/Cr and increased Glu/Cr in hippocampus of OVX chronic epileptic mice (n = 5 in each group). The data were shown as means ± SEM. Significance was calculated using ANOVA, followed by Tukey's test. *P < 0.05, **P< 0.01.

Converging lines of evidence implicate the disruption of synaptic E/I balance involved in the pathogenesis of epilepsy 13. To probe whether the ERβ-mediated enhancement of seizure susceptibility in OVX mice is dependent on synaptic E/I balance, we analyzed the expression of vesicular GABA transporter (VGAT, a marker of GABAergic neurons) and vesicular glutamate transporter 1 (VGluT1, a marker of Glutamatergic neurons) in the cortex and hippocampus of each group of OVX mice. Western blot revealed dramatically decreased VGAT expression and increased VGluT1 expression in the cortex (Figure 3A-B) and hippocampus (Figure 3C-D) from OVX chronic epileptic mice compared with OVX control mice. VGAT expression in the hippocampus of OVX control was especially decreased by deletion of ERβ. In addition, ERβ deletion typically exacerbated decrease of VGAT expression and increase of VGluT1 expression in the hippocampus of OVX chronic epileptic mice (Figure 3C-D). These results suggested that ERβ loss exacerbated synaptic E/I imbalance in the hippocampus of OVX chronic epileptic mice.

^1^H MRS is a non-invasive method to quantify brain metabolites, and ratio of N-acetyl aspartic acid/creatine (NAA/Cr) has been used to determine the lateralization of epilepsy by scanning patient's hippocampus 29. Human hippocampal ^1^H MRS data were shown in Figure 3E, and the peak of GABA, glutamic acid (Glu), and glutamine (Gln) were isolated respectively (Figure 3F). The decreased ratios of NAA/Cr, GABA/Cr and increased ratio of Glu/Cr were detected in female TLE patients compared with controls (Figure 3G).

^1^H MRS was further used to determine whether an ERβ mutation caused alterations in the endogenous metabolites of hippocampus from OVX chronic epileptic mice (Figure 3H). The peak of GABA, Glu and Gln were isolated (Figure 3I). ^1^H MRS analyses showed that the ratios of NAA/Cr, GABA/Cr and Gln/Cr were decreased and Glu/Cr was increased in the hippocampus of OVX chronic epileptic mice. And ERβ deletion greatly aggravated decreased GABA/Cr and increased Glu/Cr in OVX chronic epileptic mice (Figure 3J). Consequently, these results suggested that ERβ regulated the synaptic E/I balance in the hippocampus of OVX chronic epileptic mice, which might be involved in the promotion of epileptic seizure susceptibility.

### ERβ deletion aggravated presynaptic excitatory inputs in epileptic hippocampal CA1

**Figure 4:**
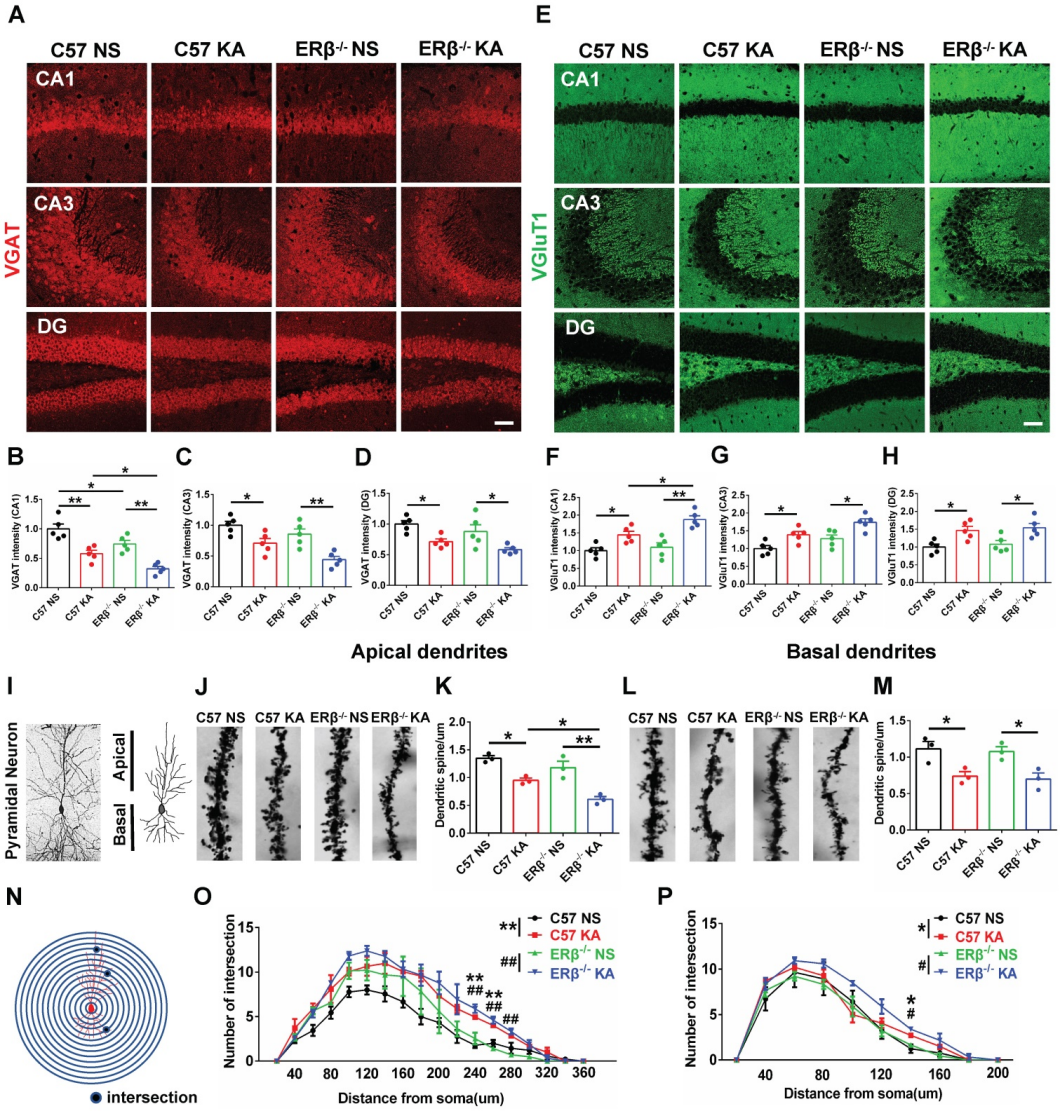
** ERβ deletion aggravated presynaptic excitatory inputs and dendrite spine loss in epileptic hippocampal CA1 pyramidal neurons. (A, E)** Immunofluorescence of VGAT (A) and VGluT1 (E) in CA1, CA3 and DG of OVX mice. Scale bars, 50 µm. **(B-D)** Immunofluorescence intensity of VGAT. **(F-H)** Immunofluorescence intensity of VGluT1. VGAT intensity were decreased and VGluT1 intensity were increased in all subregions. VGAT expression in CA1 was typically decreased by loss of ERβ. ERβ deletion especially aggravated decreased VGAT intensity and increased VGluT1 intensity in CA1 of OVX chronic epileptic mice (n = 5 in each group). **(I-M)** The density of dendritic spines was decreased in both apical and basal pyramidal neurons, and ERβ deletion further decreased the density of apical pyramidal neurons in OVX mice (n = 3 in each group). Data were shown as means ± SEM. Significance was calculated using ANOVA, followed by Tukey's test. *P < 0.05, **P< 0.01. **(N-P)** Sholl analysis showed that intersections of apical and basal CA1 pyramidal neurons were increased in OVX chronic epileptic mice (n = 3 in each group). Data were shown as means ± SEM. Significance was calculated using Two-way repeated measure ANOVA. *P < 0.05, **P< 0.01; ^#^*P* < 0.05, ^##^*P*< 0.01.

The expression levels of VGAT and VGluT1 in the different subregions of hippocampus were assessed with IF, as shown in Figure 4A and E. We found that OVX chronic epileptic mice exhibited decreased VGAT expression (Figure 4B-D) and increased VGluT1 expression (Figure 4F-H) in CA1, CA3 and dentate gyrus (DG) regions compared with OVX control mice. The VGAT intensity in CA1 region was especially decreased by loss of ERβ (Figure 4B). Importantly, ERβ deletion further downregulated VGAT and upregulated VGluT1 intensity in CA1 (Figure 4B, F) of OVX chronic epileptic mice, but not in CA3 (Figure 4C, G) and DG (Figure 4D, H). It is inferred that ERβ loss increases presynaptic excitatory inputs in the CA1 region of OVX chronic epileptic mice, causing an E/I imbalance.

### ERβ deletion exacerbated dendrite spines loss in epileptic hippocampal CA1 pyramidal neurons

Dendritic abnormalities, including complexity of dendritic branches and dendritic spine morphogenesis, are some of the most consistent neuropathological hallmarks correlated with epileptogenesis. Given the ERβ mutation typically strengthens presynaptic excitatory inputs in the CA1 of OVX chronic epileptic mice; we focused on dendrite analyses of pyramidal neurons in this region (Figure 4I). Dendrite spine density in both the apical (Figure 4J-K) and basal (Figure 4L-M) CA1 pyramidal neurons were significantly decreased in OVX chronic epileptic mice compared with OVX control mice. ERβ deletion exacerbated dendrite spine loss in the apical dendrites, but not basal dendrites (Figure 4K, M). Analysis of spine types in CA1 pyramidal neurons revealed that the proportions of mushroom spine type was decreased (Figure S5D, H) and thin spine type was increased (Figure S5F, J) in OVX chronic epileptic mice compared with OVX controls. ERβ deletion did not alter the classification of dendritic spines in OVX chronic epileptic mice. Additionally, no change in the proportion of stubby spine type was observed (Figure S5E, I).

The dendritic branch numbers of CA1 pyramidal neurons were also analyzed (Figure S5A-B). We noticed that dendritic branch numbers of apical and basal in CA1 pyramidal neurons were increased in OVX chronic epileptic mice, which was not altered by ERβ deletion (Figure S5C, G). Sholl analysis revealed a robustly enhanced dendritic complexity situated at both apical and basal dendrites in OVX chronic epileptic mice, and ERβ deletion did not affect the dendritic complexity (Figure 4N-P). These results provided evidence that ERβ loss mediated dendrite spine loss in CA1 pyramidal neuron of OVX chronic epileptic mice.

### ERβ deletion increased the frequency of sEPSC in epileptic CA1 pyramidal neurons

**Figure 5:**
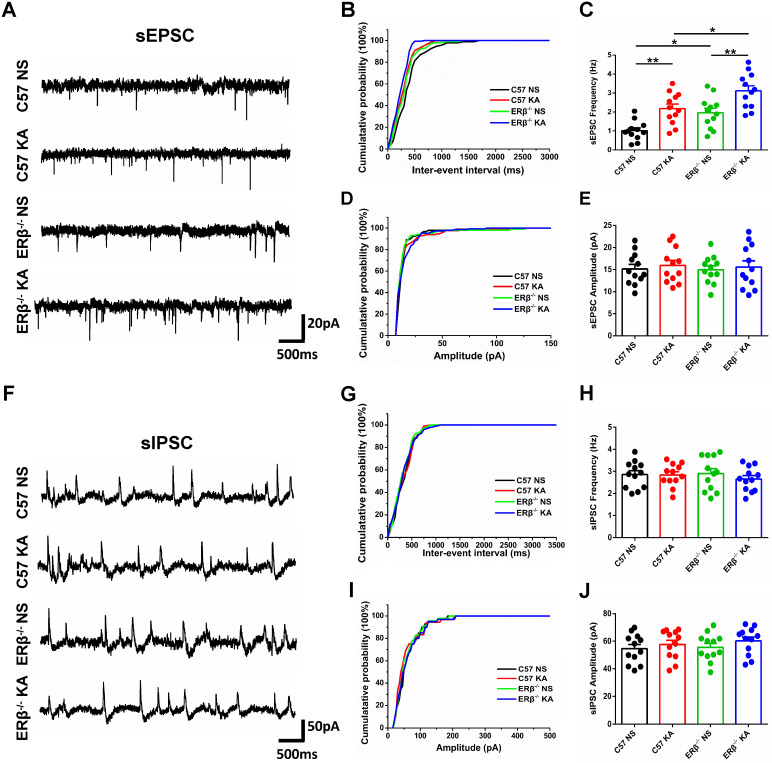
** ERβ deletion increased the sEPSC frequency of epileptic CA1 pyramidal neurons. (A)** Representative traces of sEPSCs in CA1 pyramidal neurons from OVX WT, epileptic, ERβ^-/-^ and ERβ^-/-^ epileptic mice. **(B, C)** Cumulative probability distribution and statistical comparison of sEPSC frequency. The sEPSC frequency of CA1 pyramidal neurons was significantly increased in OVX epileptic mice compared with OVX WT mice. ERβ deletion especially increased the sEPSC frequency of CA1 pyramidal neurons, and aggravated the increased sEPSC frequency in epileptic CA1 pyramidal neurons. **(D, E)** Cumulative probability distribution and statistical comparison of sEPSC amplitude. There were no differences in the sEPSC amplitude of the four groups. **(F)** Representative traces of sIPSCs.** (G, H)** Cumulative probability distribution and statistical comparison of sIPSC frequency. There were no differences in sIPSC frequency of the four groups. **(I, J)** Cumulative probability distribution and statistical comparison of sIPSC amplitude. There were no differences in sIPSC amplitude of the four groups. N = 12 cells from 4 mice in each group. Data were shown as means ± SEM. Significance was calculated using ANOVA, followed by Tukey's test. *P < 0.05, **P< 0.01.

To further investigate whether ERβ deletion affects synaptic transmission from OVX chronic epileptic mice, we performed whole-cell recordings from hippocampus CA1 pyramidal cells of OVX WT, epileptic, ERβ^-/-^ and ERβ^-/-^ epileptic mice. We found that OVX chronic epileptic mice exhibited increased sEPSC frequency in CA1 pyramidal neurons compared with OVX control mice, however, the sEPSC amplitude was unaltered. ERβ deletion also especially increased the sEPSC frequency in CA1 pyramidal neurons without any effect on the amplitude. Importantly, ERβ deletion further increased sEPSC frequency in CA1 pyramidal neurons of OVX chronic epileptic mice (Figure 5A-E). On the other hand, there were no changes in the sIPSC frequency and amplitude of CA1 pyramidal neurons from animals in four groups (Figure 5F-J). These results indicate that ERβ deletion regulates excitatory synaptic activities in CA1 pyramidal neurons.

### GLUL participated in ERβ-regulated presynaptic excitatory inputs in hippocampal CA1

**Figure 6:**
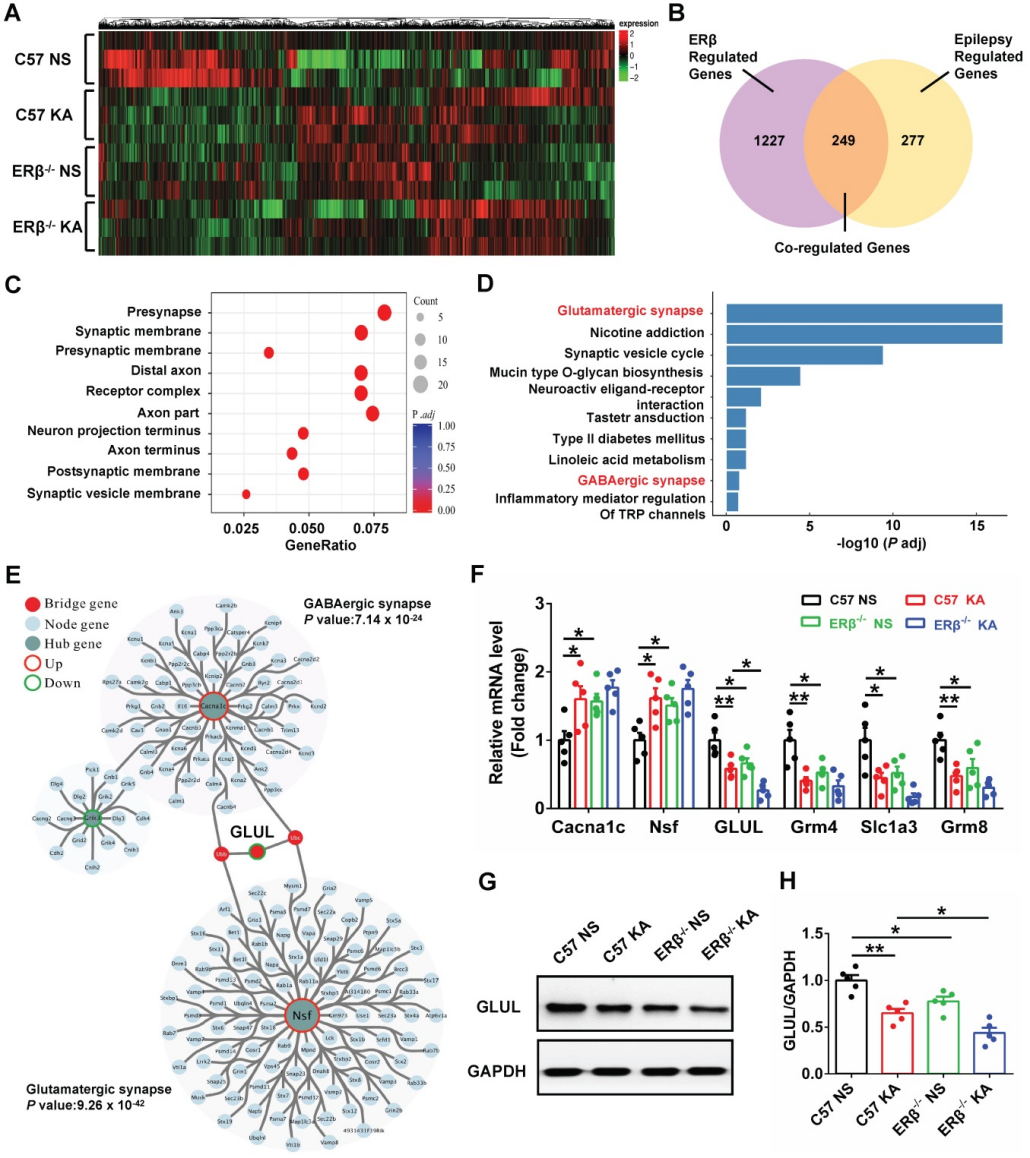
** GLUL participated in ERβ-regulated presynaptic excitatory inputs in hippocampal CA1 region. (A)** Hierarchical clustering heatmap for DEGs from the CA1 of OVX WT, epileptic, ERβ^-/-^ and ERβ^-/-^ epileptic mice (n = 3 in each group).** (B)** Venn diagram of co-regulated DEGs by epilepsy and ERβ. **(C)** Enriched top ten GO pathways in biological process.** (D)** Enriched top ten KEGG pathways in biological process. The Glutamatergic synapse and GABAergic synapse pathways were significantly changed and highlighted in red fonts. **(E)** PPI network analyses screened out GLUL as the bridge gene to link the Glutamatergic synapse and GABAergic synapse. **(F)** qRT-PCR were performed to verify the results of RNA-seq (n = 5 in each group).** (G)** Western blot of GLUL in CA1.** (H)** Western blot analysis showed that GLUL expression was decreased in OVX epileptic and ERβ^-/-^ mice compared with OVX control mice. And ERβ deletion aggravated downregulated GLUL expression in OVX epileptic mice (n = 5 in each group). Data were shown as means ± SEM. Significance was calculated using ANOVA, followed by Tukey's test. *P < 0.05, **P< 0.01.

To elucidate the potential mechanism of ERβ underlying the CA1 neuronal hyper-excitability involved in the increased seizure susceptibility of OVX mice, we performed high-throughput RNA-seq to identify genes with altered expressions. Hierarchical clustering heatmap described the gene expression profile in CA1 of OVX WT, epileptic, ERβ^-/-^ and ERβ^-/-^ epileptic mice (Figure 6A). Venn diagram showed there were 1476 ERβ regulated genes which were DEGs between OVX WT and ERβ^-/-^ mice, 526 epilepsy regulated genes which were DEGs between OVX WT and epileptic mice, and 249 genes that were co-regulated by epilepsy and ERβ among these DEGs (Figure 6B). And hierarchical clustering analysis of the 249 co-regulated genes showed the ERβ deletion had the similar effects of epilepsy on the co-regulated genes (Figure S6A). Therefore, these 249 genes played a vitally important effect in the epileptogenesis of OVX ERβ^-/-^ mice. Next, GO (Figure 6C, S6B) and KEGG (Figure 6D) enrichment were performed on these 249 DEGs. GO enrichment revealed several biological processes related to synapse structure (presynapse, synaptic membrane and presynaptic membrane) that were significantly altered (Figure 6C, S6B). In accordance, significant enrichment in KEGG pathways were found to be associated to glutamatergic synapse (Grm4, Slc1a3, Grm8 and GLUL) and GABAergic synapse (Cacna1c, Nsf and GLUL) pathways (Figure 6D). Both glutamatergic synapse and GABAergic synapse related genes were verified using qRT-PCR (Figure 6F). We noticed that ERβ deletion specially aggravated decreased GLUL mRNA level in OVX epileptic mice. GLUL, is critical in the glutamine-glutamate-GABA cycle involved in the release and uptake of glutamate and GABA.

Further, PPI network of the 249 DEGs was constructed. The genes of PPI network were divided into three groups (hub genes, bridge genes, and node genes) to categorize gene functions, and the network community recognition algorithm InfoMap was used to explore the functional relationships in the 249 DEGs. Two communities (glutamatergic synapse and GABAergic synapse community) were detected with community nodes ≥ 15 and an adj. p-value < 0.01. GLUL, connecting the two communities, was considered as the bridge gene regulating both the glutamatergic and GABAergic synapse (Figure 6E). Therefore, we inferred that GLUL contributed to ERβ-regulated presynaptic excitatory inputs in hippocampal CA1. In addition, the protein levels of GLUL in the CA1 region were detected, and significantly decreased GLUL levels were found in OVX chronic epileptic and ERβ^-/-^ mice compared with OVX control mice. And ERβ deletion especially decreased protein level of GLUL in OVX epileptic mice (Figure 6G-H). These results indicate that increased susceptibility to KA-induced epilepsy in OVX ERβ^-/-^ mice could be linked to altered GLUL level in the CA1.

### WAY alleviated spontaneous seizures and inhibition of GLUL expression

**Figure 7:**
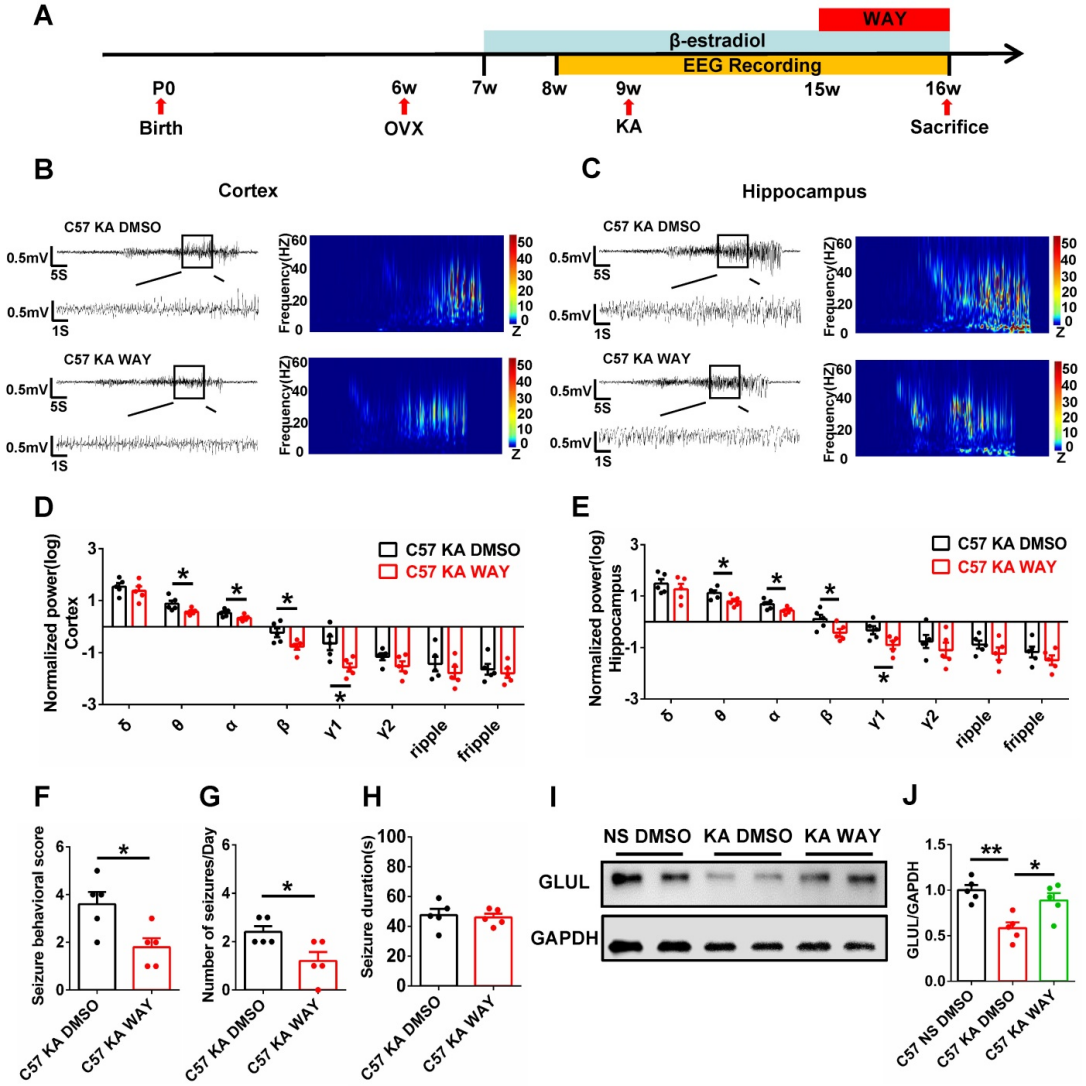
** WAY-200070 alleviated spontaneous seizures and decreased GLUL expression in OVX epileptic mice. (A)** Diagram of the experimental paradigm. **(B**, **C)** Representative EEG recordings of the cortex (B) and hippocampus (C) from OVX chronic epileptic and WAY-treated OVX chronic epileptic mice.** (D**, **E)** Normalized EEG power in cortex (D) and hippocampus (E). WAY treatment decreased θ, α, β and γ1 oscillations of cortex and hippocampus from OVX chronic epileptic mice. **(F**-**H)** Seizure behavioral score (F), number of seizures per day (G) and seizure duration (H) of OVX chronic epileptic and WAY-treated OVX chronic epileptic mice. WAY treatment decreased the seizure behavioral score and number of seizures per day, but it did not change the average seizure duration.** (I)** Western blot of GLUL in the CA1 of OVX WT, chronic epileptic and WAY-treated OVX chronic epileptic mice.** (J)** Western blot analysis showed that GLUL expression was decreased in CA1 of OVX chronic epileptic mice, and WAY treatment significantly rescued the decreased GLUL. N = 5 in each group. Data were shown as means ± SEM. Significance was calculated using ANOVA, followed by Tukey's test. *P < 0.05, **P< 0.01.

The antiepileptogenic effects of ERβ agonist WAY were also investigated. OVX epileptic mice with SRS were s.c. daily injected with castor oil containing vehicle (DMSO) or WAY (10 mg/kg) for one week (Figure 7A) at 15 weeks of age 30. EEG recordings of the cortex and hippocampus from OVX chronic epileptic and WAY-treated OVX chronic epileptic mice were shown in Figure 7B and C. Normalized EEG power were analyzed, and results revealed that WAY treatment downregulated θ, α, β and γ1 oscillation in cortex (Figure 7D) and hippocampus (Figure 7E). Further, WAY treatment decreased seizure behavioral score (Figure 7F) and seizures frequency (Figure 7G), without alteration in average seizure duration of OVX chronic epileptic mice (Figure 7H). In addition, the protein level of GLUL was decreased in CA1 of OVX chronic epileptic mice, which could be rescued by WAY treatment (Figure 7I-J). These results showed that WAY attenuates the SRS induced by KA and have a potentially protective role in OVX epileptic mice.

## Discussion

The present study identifies a previously unknown role of the ERβ in females with TLE. We detected decreased levels of ERβ expression in the brain tissues of both female TLE patients and OVX chronic epileptic mice. Remarkably, ERβ deletion in OVX mice increased seizure susceptibility and exacerbated imbalance of synaptic E/I in the CA1 region. To begin to understand how ERβ downregulation may cause TLE, RNA-seq was performed to identify ERβ target RNAs. Remarkably, GLUL was involved in the regulation of seizure susceptibility in OVX mice, supplementation with ERβ agonist WAY rescued epileptic seizures and decreased GLUL expression. Our results identify ERβ as an important therapeutic target for the management of epilepsy.

Increasing evidence based on observations made from experimental animals and human patients suggests that estrogens produced the proconvulsant effects and may increase the severity of epilepsy 4. Our experiments revealed decreased ERβ expression in the temporal neocortex and hippocampus of female TLE subjects, similarly to that observed in the chronic epileptic mouse model, suggesting that down-regulation of ERβ plays a role in the development of human epilepsy. Although it is not clear that ERβ deletion participates in the chain of events involved in the epileptogenesis, our animal studies showed that behavioral seizure scores were higher and pathological changes caused by epilepsy were more serious in OVX ERβ^-/-^ chronic epileptic mice, indicating that ERβ deletion can increase susceptibility to seizures.

It is widely believed that the disruption of synaptic E/I balance is a potential mechanism of epileptogenesis 13, 31. Multiple studies have identified either loss of GABAergic inhibition or activation of glutamatergic function or both in human epileptic brain. Status epilepticus induced glutamate release sustained long-term seizure activity, and N-methyl-d-aspartate receptor (NMDARs) antagonist suppressed epileptic seizures after prolonged status epilepticus 32, 33. In addition, reduction of GABAergic synapses existed in surviving neurons of epileptic brains, and breakdown of neuronal inhibition was vital for seizure propagation in epileptic models 34. Chronic epileptic mouse model used in this study produced SE via intra-hippocampus KA microinjection, epileptiform activity starting from the hippocampus (the site of injection) and then successively propagating to the cortex that is highly isomorphic to human TLE 35. Here, we found OVX WT and ERβ^-/-^ chronic epileptic mice displayed significantly reduced VGAT and increased VGluT1 in the cortex and hippocampus. Notably, ERβ deletion exacerbated the damage of synaptic E/I balance especially in hippocampus but not cortex of OVX chronic epileptic mice, which might explain that hippocampus was prone to the generation of epileptiform activity and seizures. As expected, ERβ deletion exacerbated the decreased GABA/Cr and increased Glu/Cr in the hippocampus of OVX epileptic mice compared to WT OVX epileptic mice. The similar alterations in the GABA/Cr and Glu/Cr were also detected in female TLE hippocampus. These findings highlight aggravating deficit of synaptic E/I balance in hippocampus induced by ERβ deletion is likely to be a key factor in the pathogenesis of epileptogenesis.

Hippocampus is the primary susceptible region in epileptic pathology, and hippocampal sclerosis is the commonest cause of drug-resistant epilepsy in adults 36. Typical histopathologic changes of epileptic hippocampus were found in OVX chronic epileptic mice, including gliosis, neuronal loss and mossy fibers sprouting 37. Furthermore, pathologies in hippocampus generate increased seizure susceptibility and cognitive deficits in TLE 38. ERβ is abundantly expressed in hippocampus and reported to be involved in the protection of neurogenesis and cognitive function 39. In this study, we found ERβ expression in hippocampus was positively correlated with Wechsler intelligence score of female TLE patients, and ERβ deletion greatly aggravated pathologic changes in hippocampus of OVX chronic epileptic mice.

CA1 region is the final output of hippocampus which can receive glutamatergic input from CA3 40. It is associated with an increase in activity prior to seizure activity and generally impaired in TLE 41. In humans with intractable partial-onset epilepsy and experimental models of epilepsy, CA1 pyramidal neurons show persistent hyperexcitability and neuronal loss 42. Our previous studies showed that ERβ agonist LY3201 elevated GABAergic signaling in CA1 17, and we found ERβ deletion aggravated the synaptic E/I imbalance in CA1 of OVX chronic epileptic mice in this study. Dendritic formation and maturation regulate the neuronal excitability, and dendritic abnormalities in CA1 pyramidal neurons have been reported in pathological tissue specimens from epilepsy patients and mice 43. We observed that there were decreased dendritic spine density, increased dendritic complexity, and smaller proportion of mature mushroom-shaped and greater immature thin-shaped dendritic spines in CA1 pyramidal neurons of OVX chronic epileptic mice. And ERβ deletion further decreased apical dendritic density of CA1 pyramidal neurons, indicating that ERβ deletion worsened dendritic abnormalities of CA1 pyramidal neurons. Furthermore, we found that ERβ deletion augmented sEPSC frequency in CA1 pyramidal neurons of OVX chronic epileptic mice, implies that this increased synaptic activity. Therefore, ERβ is involved in epileptogenesis of female patients with epilepsy mainly through affecting hyperexcitability of CA1 pyramidal neurons.

The target genes regulated by ERβ involved in synaptic E/I imbalance of OVX epileptic mice were explored through high throughput RNA-seq. DEGs co-regulated by ERβ (OVX ERβ^-/-^ vs WT mice) and epilepsy (OVX epileptic vs WT mice) were screened, and significant differences of presynaptic and postsynaptic functions were enriched by GO terms, and glutamatergic and GABAergic synapse were enriched by KEGG pathways. PPI network analyses selected the GLUL as the bridge gene to link the Glutamatergic and GABAergic synapse. GLUL, is critical in the glutamine-glutamate-GABA cycle which ensures the fidelity of excitatory and inhibitory synaptic transmission, the release and uptake of glutamate and GABA 44. GLUL is severely deficient in hippocampus of patients with mesial temporal lobe epilepsy (MTLE), and surgically resected brain tissue from MTLE showed 40% loss of GLUL protein and enzyme activity 45. Notably, GLUL deficiency downregulated GABA and glutamine levels, and upregulated glutamate levels in epileptic brains 46. As expected, ^1^H MRS examination of female patients and OVX mice with epilepsy showed decreased GABA/Cr and increased Glu/Cr in the hippocampus. Meanwhile, Gln/Cr represented significant reduction in OVX chronic epileptic mice. In addition, the GLUL expression was confirmed to be decreased in OVX epileptic mice compared to controls, and ERβ deletion aggravated the decreased expression of GLUL in OVX epileptic mice. Consistent with our previous study 17, we further confirmed that ERβ agonist WAY treatment rescued epileptic seizures and alleviated decreased GLUL expression in CA1 of OVX chronic epileptic mice. These results indicated that ERβ deletion worsened the imbalance of E/I in OVX chronic epileptic mice through regulating GLUL expression, and WAY treatment may have potential antiepileptic effects by rescuing downregulated GLUL expression. In addition, further investigations are needed to unveil the precise mechanism behind ERβ-mediated regulation of GLUL in female epilepsy.

In conclusion, our study demonstrates downregulated ERβ increases the susceptibility to epileptic seizures and aggravates the imbalance between neuronal excitability and inhibition of CA1 in female epilepsy. ERβ activation alleviates epileptic activity and decreases GLUL expression in female epilepsy. Clinical application of ERβ agonist in female patients with TLE needs to be further investigated. Taken together, our data reveal a novel antiepileptic target to female patients with epilepsy.

## Supplementary Material

Supplementary figures and tables.

## Abbreviations

AED: antiepileptic drug
DEGs: differential expression genes
EEG: electroencephalogram
E/I: excitation/inhibition
ERβ: estrogen receptor β
GLUL: glutamine ligase
GO: gene ontology
^1^H MRS: proton magnetic resonance spectroscopy
IF: immunofluorescence
IHC: immunohistochemistry
KA: kainic acid
KEGG: kyoto encyclopedia of genes and genomes
VGAT: vesicular GABA transporter
VGluT1: vesicular glutamate transporter 1
OVX: ovariectomized
PPI: protein-protein interaction
qRT-PCR: quantitative real-time polymerase chain reaction
SRS: spontaneous recurrent seizures
TLE: female temporal lobe epilepsy
WAY: WAY-200070
