# Supplementary material for: Female mice lacking ERβ display excitatory/inhibitory synaptic imbalance to drive the pathogenesis of temporal lobe epilepsy
Source: Theranostics. 2021 Apr 7;11(12):6074–89. doi: 10.7150/thno.56331 (PMC8058727; doi:10.7150/thno.56331)
Supplement: Supplementary file 1 — Supplementary figures and tables. [file thnov11p6074s1.pdf]

Supplementary Materials for “Female mice lacking estrogen receptor β display excitatory/inhibitory synaptic imbalance to drive the pathogenesis of temporal lobe epilepsy”.

Figure S1. Correlation analyses of ERβ expression and clinical features in female TLE patients.

Figure S2. ERβ expression was down-regulated in OVX chronic epileptic mice.

Figure S3. ERβ deletion increased seizure susceptibility in OVX acute epileptic mice.

Figure S4. ERβ deletion exacerbated pathological changes of OVX chronic epileptic mice.

Figure S5. ERβ deletion did not affect dendritic branches and classification in CA1 pyramidal neurons of OVX chronic epileptic mice.

Figure S6. Hierarchical clustering and GO enrichment analysis for DEGs co-regulated by ERβ and epilepsy.

Table S1. Clinical features of female patients with TLE.

Table S2. Clinical features of control subjects.

Table S3. The sequences of qRT-PCR primers used in this study.

Figure S1

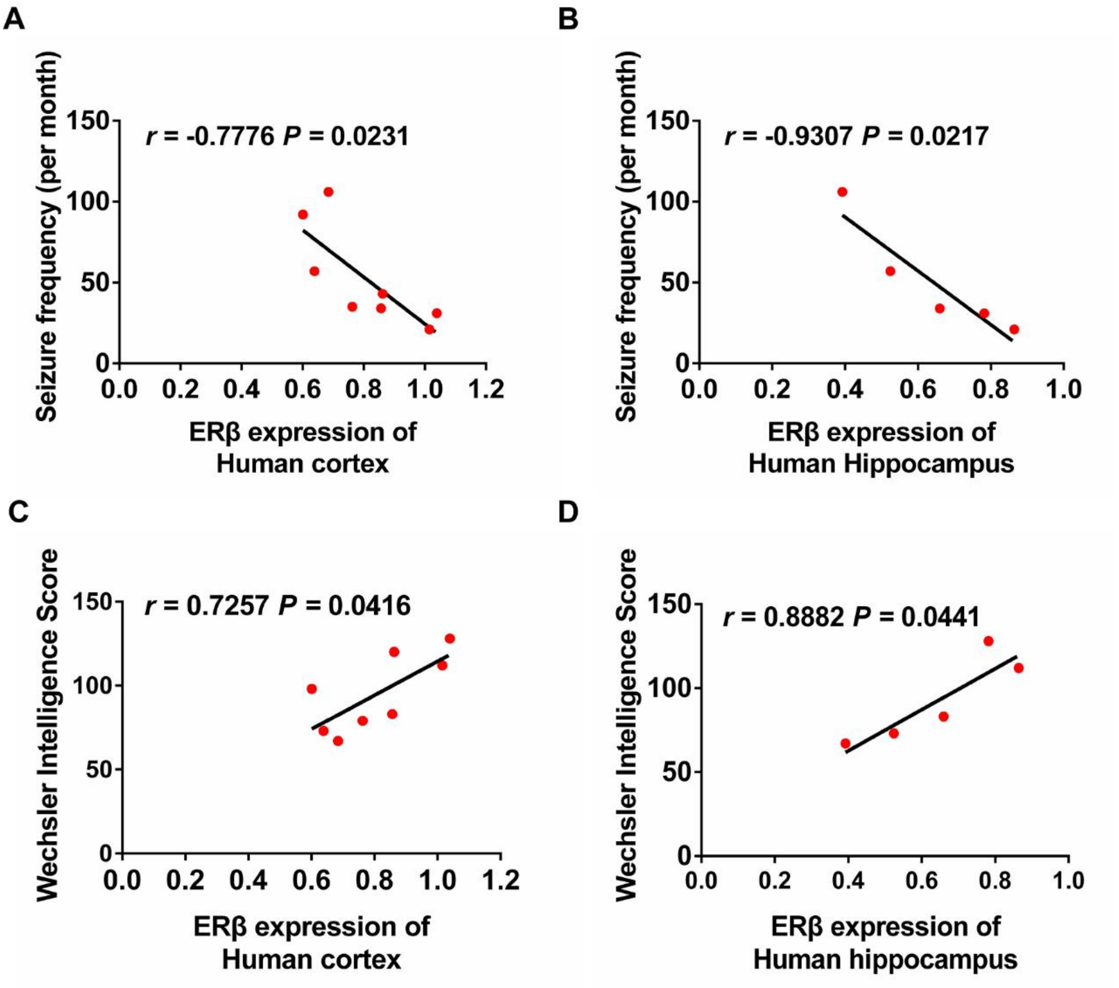

Figure S1. Correlation analyses of ERβ expression and clinical features in female TLE patients. (A, **B)** Spearman rank correlation between ERβ expression and seizure frequency in temporal neocortex (A) and hippocampus (B) of female TLE patients. There were negative correlations between ERβ expression and seizure frequency in temporal neocortex (n = 8, *r* = -0.7776, *P* = 0.0231) and hippocampus (n = 5, *r* = -0.9307, *P* = 0.0217) of female TLE patients. **(C**, **D)** Spearman rank correlation between ERβ expression and Wechsler intelligence scores in temporal neocortex (C) and hippocampus (D) of female TLE patients. There were positive correlations between ERβ expression and Wechsler intelligence score in temporal neocortex (n = 8,

r = 0.7257, P = 0.0416) and hippocampus (n = 5, r = 0.8882, P = 0.0441) of female TLE patients.

Figure S2

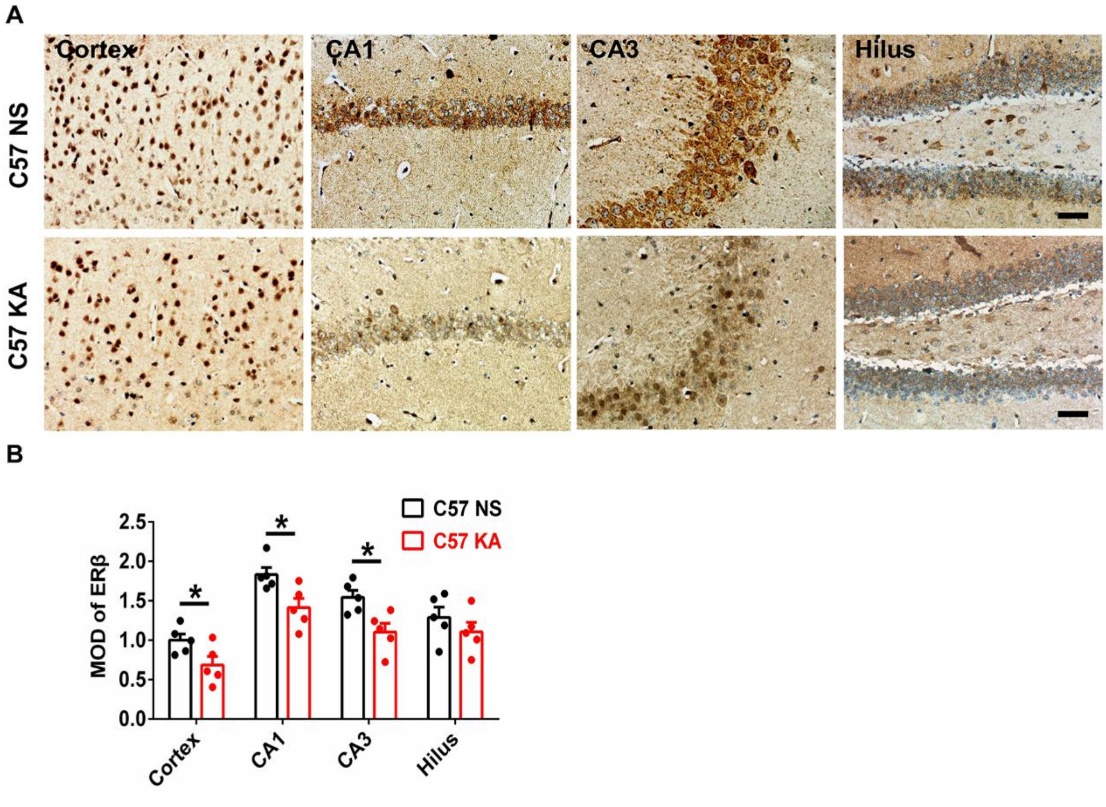

Figure S2. ERβ expression was down-regulated in OVX chronic epileptic mice. **(A)** Immunostaining of ERβ in the cortex and hippocampus of OVX chronic epileptic mice and OVX control mice (n = 5 in each group). Scale bars, 50 µm. **(B)** Quantification of ERβ in immunostaining. MOD of ERβ was decreased in the cortex, CA1 and CA3 regions of OVX chronic epileptic mice compared with controls. The data were shown as means ± SEM. Significance was calculated using ANOVA, followed by Tukey's test. *P < 0.05.

Figure S3

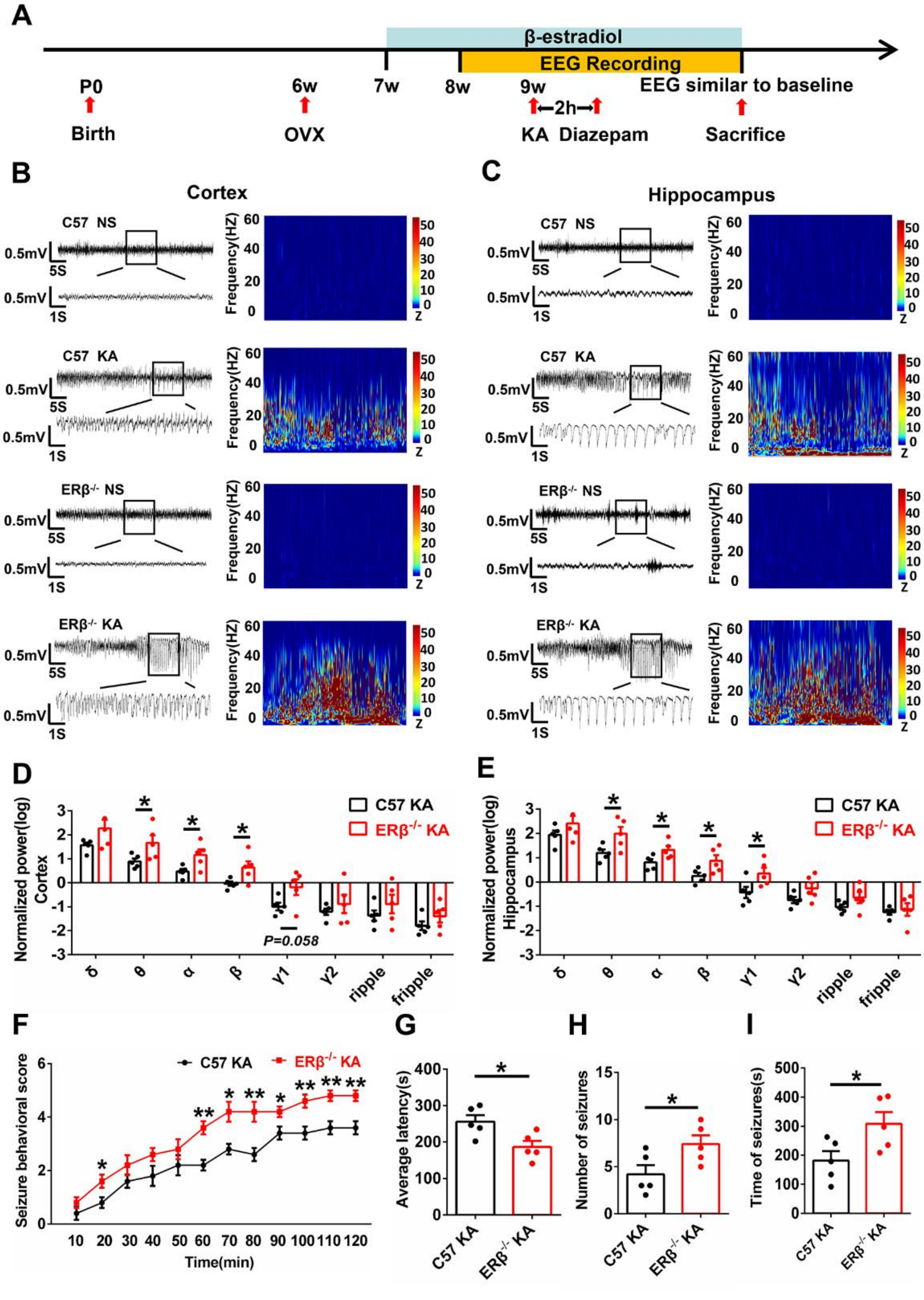

Figure S3. ERβ deletion increased seizure susceptibility in OVX acute epileptic mice. (A) Diagram of experimental paradigm. **(B, C)** Representative basal and acute epileptic EEG recordings from the cortex (B) and hippocampus (C) of OVX mice. **(D, E)** Spectral analysis of acute epileptic EEG recordings. ERβ deletion increased θ, α, β oscillations in cortex (D) and θ, α, β, γ1 oscillations in hippocampus (E) of OVX

acute epileptic mice. **(F-I)** Seizure behavioral score (F), average latency (G), number (H) and time (I) of seizures before SE in OVX acute epileptic mice. ERβ deletion increased seizure behavioral scores, number, and time of seizures before SE, and decreased the latency of acute seizures. N = 5 in each group. The data were shown as means ± SEM. Significance was calculated using Student's *t* test. $*P < 0.05$, $**P < 0.01$.

Figure S4

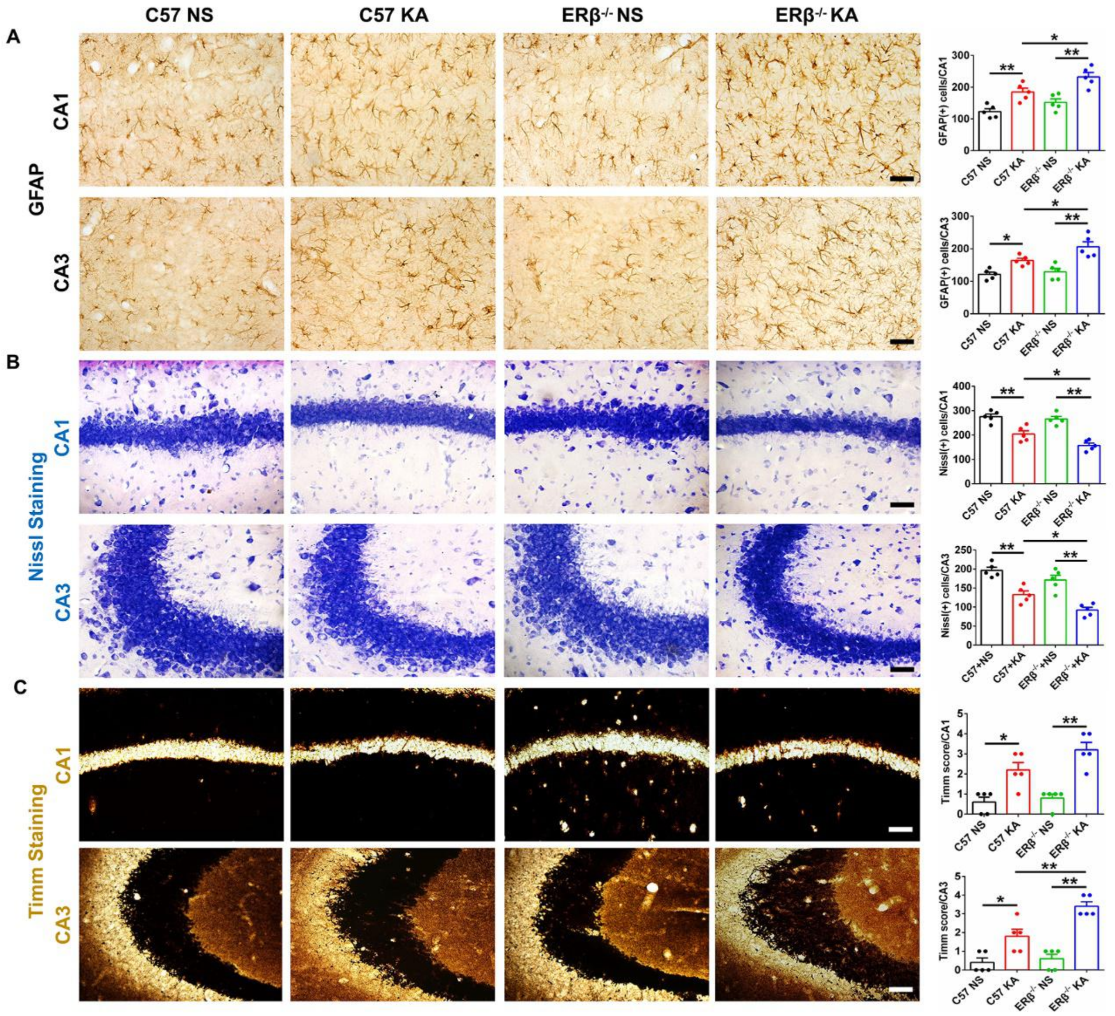

Figure S4. ERβ deletion exacerbated pathological changes of OVX chronic epileptic mice. (A) Immunostaining of GFAP in the CA1 and CA3 regions of each group of OVX mice. ERβ deletion aggravated the glosis in the CA1 and CA3 regions

of OVX chronic epileptic mice. **(B)** Nissl staining in the CA1 and CA3 regions of each group of OVX mice. ERβ deletion aggravated the neuron loss of the CA1 and CA3 regions in OVX chronic epileptic mice. **(C)** Timm staining in the CA1 and CA3 regions of each group of OVX mice. ERβ deletion aggravated moss fibers sprouting in the CA3 region of OVX chronic epileptic mice, but did not affect moss fibers sprouting in the CA1 region. Scale bars, 50 μm. N = 5 in each group. The data were shown as means ± SEM. Significance was calculated using ANOVA, followed by Tukey's test. *$P < 0.05$, **$P < 0.01$.

Figure S5

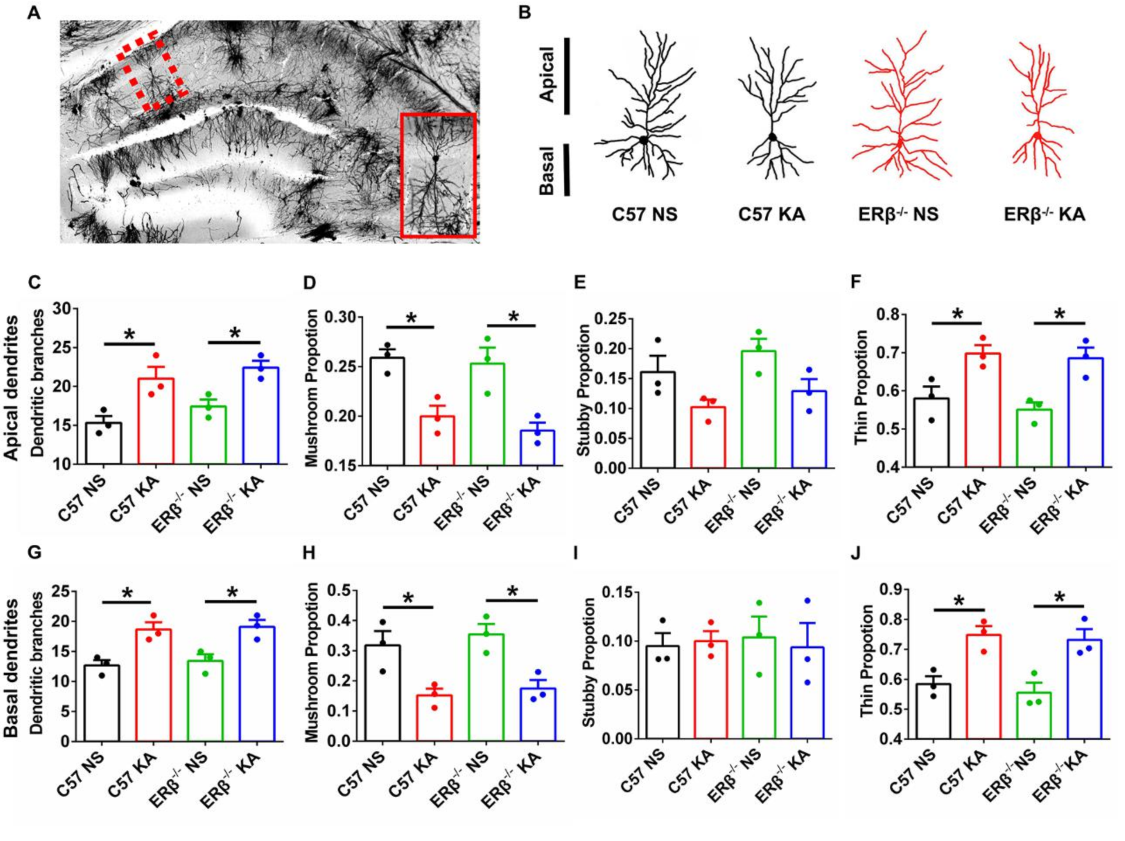

Figure S5. ERβ deletion did not affect dendritic branches and classification in CA1 pyramidal neurons of OVX chronic epileptic mice. (A, B) Representative image (A) and reconstruction (B) of CA1 pyramidal neurons from OVX mice (n = 3

in each group). **(C, G)** Dendritic branches of CA1 pyramidal neurons. Both of apical (C) and basal (G) dendritic branches were decreased in CA1 pyramidal neurons of OVX chronic epileptic mice, but ERβ deletion did not affect the dendritic branches of CA1 pyramidal neurons. **(D-F)** Classification of dendritic spines in CA1 apical pyramidal neurons. **(H-J)** Classification of dendritic spines in CA1 basal pyramidal neurons. The proportion of mushroom dendritic spines was decreased and the proportion of thin dendritic spines were increased in CA1 pyramidal neurons of OVX chronic epileptic mice compared with OVX controls. ERβ deletion did not change the classification of dendritic spines in CA1 pyramidal neurons from OVX mice. There were no changes in the proportion of stubby dendritic spines in CA1 pyramidal neurons from OVX mice. The data were shown as means ± SEM. Significance was calculated using ANOVA, followed by Tukey's test. *$P < 0.05$, **$P < 0.01$.

Figure S6

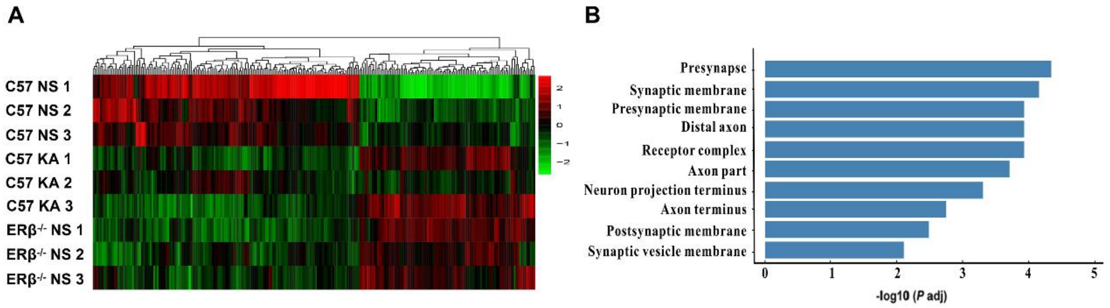

Figure S6. Hierarchical clustering and GO enrichment analysis for DEGs co-regulated by ERβ and epilepsy. (A) Hierarchical clustering analysis of co-regulated DEGs. **(B)** GO enrichment analysis of co-regulated DEGs.

Table S1. Clinical features of female patients with TLE.

Case No.	Gender	Diagnosis	Age at surgery (year)	Epilepsy duration (year)	Seizure frequency (month)	Seizure type	Location of surgical resection	Engel's class	Application in this study
1	F	TLE	48	6.5	45	PS;	L temporal	II	IHC,
2	F	TLE	34	2	72	PS; IS;	R temporal	II	IHC,
3	F	TLE	58	2.5	57	PS;	R temporal, R hippocampus	I	WB, IHC,
4	F	TLE	23	5	21	PS;	L temporal	I	IHC,
5	F	TLE	31	4	42	GTCS;	R temporal	I	IHC,
6	F	TLE	33	4	106	PS; GTCS;	L temporal, L hippocampus	II	WB, IHC,
7	F	TLE	23	3	45	PS;	L temporal	I	IHC,
8	F	TLE	64	1	57	PS;	L temporal, L hippocampus	I	IHC,
9	F	TLE	54	8	92	GTCS;	R temporal	I	WB,
10	F	TLE	51	7	34	PS; Tonic;	L temporal	II	WB, IHC,
11	F	TLE	40	5.5	22	GTCS;	L temporal	I	IHC,
12	F	TLE	29	3.5	43	PS;	R temporal, R hippocampus	I	WB,
13	F	TLE	27	2	27	PS;	R temporal	II	IHC,
14	F	TLE	65	5.5	57	PS;	L temporal	III	IHC,
15	F	TLE	46	3	35	GTCS; Tonic;	R temporal, R hippocampus	II	WB, IHC,
16	F	TLE	33	4	21	GTCS;	L temporal	I	WB,
17	F	TLE	59	2	31	GTCS;	L temporal, L hippocampus	I	WB, IHC,

F, Female; TLE, temporal lobe epilepsy; PS, partial seizure; IS, infantile spasm; GTCS, generalized tonic-clonic seizure; Tonic, tonic seizure; WB, Western blot; IHC, immunohistochemistry

Table S2. Clinical features of control subjects.

Case No.	Gender	Age (year)	Cause of death	PMI (h)	Location of resection	Seizure	Application in the present study
1	F	16	Motor vehicle accident	2.0	L temporal; L hippocampus	None	WB, IHC
2	F	41	Non-neurological disease	3.0	R temporal	None	IHC
3	F	27	Non-neurological disease	4.5	L temporal	None	WB
4	F	36	Motor vehicle accident	2.2	L temporal; L hippocampus	None	WB, IHC
5	F	45	Non-neurological disease	3.6	R temporal	None	WB, IHC,
6	F	57	Non-neurological disease	2.1	L temporal	None	WB,
7	F	22	Motor vehicle accident	1.7	R temporal; R hippocampus	None	WB, IHC

8	F	38	Non-neurological disease	3.0	R temporal	None	IHC
9	F	47	Non-neurological disease	2.8	L temporal	None	WB, IHC
10	F	32	Motor vehicle accident	2.4	L temporal	None	IHC
11	F	56	Non-neurological disease	4.0	R temporal	None	IHC
12	F	16	Non-neurological disease	3.5	L temporal	None	WB, IHC

Table S3. The sequences of qRT-PCR primers used in this study.

Gene name	Forward	Reverse
Grm4	CCCATACCCATTGTCAAGTTGG	TGTAGCGCACAAAAGTGACCA
Slcla3	ACCAAAAGCAACGGAGAAGAG	GGCATTCCGAAACAGGTAACTC
Grm8	ATGGTTTGTGAGGGAAAGCG	GAATGGGCATACTCCTGGCT
GLUL	TGAACAAAGGCATCAAGCAAATG	CAGTCCAGGGTACGGGTCTT
Cacna1c	ATGAAAACACGAGGATGTACGTT	ACTGACGGTAGAGATGGTTGC
Nsf	CGGACTATGCAAGCTGCGA	AACCGCACAGTTGCTTAAAGA
GAPDH	AGGTCGGTGTGAACGGATTTG	GGGGTCGTTGATGGCAACA
